# Respiratory Viral Infections in Exacerbation of Chronic Airway Inflammatory Diseases: Novel Mechanisms and Insights From the Upper Airway Epithelium

**DOI:** 10.3389/fcell.2020.00099

**Published:** 2020-02-25

**Authors:** Kai Sen Tan, Rachel Liyu Lim, Jing Liu, Hsiao Hui Ong, Vivian Jiayi Tan, Hui Fang Lim, Kian Fan Chung, Ian M. Adcock, Vincent T. Chow, De Yun Wang

**Affiliations:** ^1^Department of Otolaryngology, Yong Loo Lin School of Medicine, National University of Singapore, Singapore, Singapore; ^2^Infectious Disease Research and Training Office, National Centre for Infectious Diseases, Singapore, Singapore; ^3^Division of Respiratory and Critical Care Medicine, National University Hospital, Singapore, Singapore; ^4^Department of Medicine, Yong Loo Lin School of Medicine, National University of Singapore, Singapore, Singapore; ^5^Airway Disease, National Heart and Lung Institute, Faculty of Medicine, Imperial College London, London, United Kingdom; ^6^Department of Microbiology and Immunology, Yong Loo Lin School of Medicine, National University of Singapore, Singapore, Singapore

**Keywords:** chronic airway inflammatory diseases, respiratory virus, acute exacerbation, upper airway, epithelium

## Abstract

Respiratory virus infection is one of the major sources of exacerbation of chronic airway inflammatory diseases. These exacerbations are associated with high morbidity and even mortality worldwide. The current understanding on viral-induced exacerbations is that viral infection increases airway inflammation which aggravates disease symptoms. Recent advances in *in vitro* air-liquid interface 3D cultures, organoid cultures and the use of novel human and animal challenge models have evoked new understandings as to the mechanisms of viral exacerbations. In this review, we will focus on recent novel findings that elucidate how respiratory viral infections alter the epithelial barrier in the airways, the upper airway microbial environment, epigenetic modifications including miRNA modulation, and other changes in immune responses throughout the upper and lower airways. First, we reviewed the prevalence of different respiratory viral infections in causing exacerbations in chronic airway inflammatory diseases. Subsequently we also summarized how recent models have expanded our appreciation of the mechanisms of viral-induced exacerbations. Further we highlighted the importance of the virome within the airway microbiome environment and its impact on subsequent bacterial infection. This review consolidates the understanding of viral induced exacerbation in chronic airway inflammatory diseases and indicates pathways that may be targeted for more effective management of chronic inflammatory diseases.

## Search Strategy

Search performed between July to November 2019, results is as of 15th November 2019

1. (virus OR viral) AND (chronic airway inflammat^∗^ OR airway inflamma^∗^ OR inflammat^∗^ OR asthma OR rhinosinusitis OR COPD OR Chronic Obstructive Pulmonary Disease) **61513 results**
2. (viral OR virus) AND (asthma OR rhinosinusitis OR COPD OR chronic obstructive pulmonary disease chronic OR chronic OR inflammation OR chronic inflammation) AND (airway OR lung OR nose OR nasal OR upper airway OR lower airway) **10622 results**
3. (virus OR viral) AND (chronic airway inflammat^∗^ OR airway inflamma^∗^ OR inflammat^∗^ OR asthma OR rhinosinusitis OR COPD OR Chronic Obstructive Pulmonary Disease) AND (epitheli^∗^) **5029 results**
4. (viral OR virus) AND exacerbation AND (asthma OR rhinosinusitis OR COPD OR chronic obstructive pulmonary disease OR chronic OR inflammation OR airway) **1916 results**
5. (viral OR virus) AND exacerbation AND (asthma OR rhinosinusitis OR COPD OR chronic obstructive pulmonary disease chronic OR chronic OR inflammation OR chronic inflammation) AND (airway OR lung OR nose OR nasal OR upper airway OR lower airway) **641 results**
6. (viral OR virus) AND exacerbation AND (asthma OR rhinosinusitis OR COPD OR chronic obstructive pulmonary disease OR chronic OR inflammation OR airway) AND (epitheli^∗^) **177 results**
7. (viral OR virus) AND exacerbation AND (asthma OR rhinosinusitis OR COPD OR chronic obstructive pulmonary disease chronic OR chronic OR inflammation OR chronic inflammation) AND (airway OR lung OR nose OR nasal OR upper airway OR lower airway) AND (epitheli^∗^) **150 results**.

Additional literature was retrieved from citations within the articles of interest.

Article selection was performed with a focus on works from years 2009 to 2019.

## Introduction

The prevalence of chronic airway inflammatory disease is increasing worldwide especially in developed nations (GBD 2015 Chronic Respiratory Disease Collaborators, 2017; Guan et al., 2018). This disease is characterized by airway inflammation leading to complications such as coughing, wheezing and shortness of breath. The disease can manifest in both the upper airway (such as chronic rhinosinusitis, CRS) and lower airway (such as asthma and chronic obstructive pulmonary disease, COPD) which greatly affect the patients’ quality of life (Calus et al., 2012; Bao et al., 2015). Treatment and management vary greatly in efficacy due to the complexity and heterogeneity of the disease. This is further complicated by the effect of episodic exacerbations of the disease, defined as worsening of disease symptoms including wheeze, cough, breathlessness and chest tightness (Xepapadaki and Papadopoulos, 2010). Such exacerbations are due to the effect of enhanced acute airway inflammation impacting upon and worsening the symptoms of the existing disease (Hashimoto et al., 2008; Viniol and Vogelmeier, 2018). These acute exacerbations are the main cause of morbidity and sometimes mortality in patients, as well as resulting in major economic burdens worldwide. However, due to the complex interactions between the host and the exacerbation agents, the mechanisms of exacerbation may vary considerably in different individuals under various triggers.

Acute exacerbations are usually due to the presence of environmental factors such as allergens, pollutants, smoke, cold or dry air and pathogenic microbes in the airway (Gautier and Charpin, 2017; Viniol and Vogelmeier, 2018). These agents elicit an immune response leading to infiltration of activated immune cells that further release inflammatory mediators that cause acute symptoms such as increased mucus production, cough, wheeze and shortness of breath. Among these agents, viral infection is one of the major drivers of asthma exacerbations accounting for up to 80–90% and 45–80% of exacerbations in children and adults respectively (Grissell et al., 2005; Xepapadaki and Papadopoulos, 2010; Jartti and Gern, 2017; Adeli et al., 2019). Viral involvement in COPD exacerbation is also equally high, having been detected in 30–80% of acute COPD exacerbations (Kherad et al., 2010; Jafarinejad et al., 2017; Stolz et al., 2019). Whilst the prevalence of viral exacerbations in CRS is still unclear, its prevalence is likely to be high due to the similar inflammatory nature of these diseases (Rowan et al., 2015; Tan et al., 2017). One of the reasons for the involvement of respiratory viruses’ in exacerbations is their ease of transmission and infection (Kutter et al., 2018). In addition, the high diversity of the respiratory viruses may also contribute to exacerbations of different nature and severity (Busse et al., 2010; Costa et al., 2014; Jartti and Gern, 2017). Hence, it is important to identify the exact mechanisms underpinning viral exacerbations in susceptible subjects in order to properly manage exacerbations via supplementary treatments that may alleviate the exacerbation symptoms or prevent severe exacerbations.

While the lower airway is the site of dysregulated inflammation in most chronic airway inflammatory diseases, the upper airway remains the first point of contact with sources of exacerbation. Therefore, their interaction with the exacerbation agents may directly contribute to the subsequent responses in the lower airway, in line with the “United Airway” hypothesis. To elucidate the host airway interaction with viruses leading to exacerbations, we thus focus our review on recent findings of viral interaction with the upper airway. We compiled how viral induced changes to the upper airway may contribute to chronic airway inflammatory disease exacerbations, to provide a unified elucidation of the potential exacerbation mechanisms initiated from predominantly upper airway infections.

## Significance of Virus Infection in Exacerbation of Chronic Airway Inflammatory Diseases

Despite being a major cause of exacerbation, reports linking respiratory viruses to acute exacerbations only start to emerge in the late 1950s (Pattemore et al., 1992); with bacterial infections previously considered as the likely culprit for acute exacerbation (Stevens, 1953; Message and Johnston, 2002). However, with the advent of PCR technology, more viruses were recovered during acute exacerbations events and reports implicating their role emerged in the late 1980s (Message and Johnston, 2002). Rhinovirus (RV) and respiratory syncytial virus (RSV) are the predominant viruses linked to the development and exacerbation of chronic airway inflammatory diseases (Jartti and Gern, 2017). Other viruses such as parainfluenza virus (PIV), influenza virus (IFV) and adenovirus (AdV) have also been implicated in acute exacerbations but to a much lesser extent (Johnston et al., 2005; Oliver et al., 2014; Ko et al., 2019). More recently, other viruses including bocavirus (BoV), human metapneumovirus (HMPV), certain coronavirus (CoV) strains, a specific enterovirus (EV) strain EV-D68, human cytomegalovirus (hCMV) and herpes simplex virus (HSV) have been reported as contributing to acute exacerbations (Zheng et al., 2018). The common feature these viruses share is that they can infect both the upper and/or lower airway, further increasing the inflammatory conditions in the diseased airway (Mallia and Johnston, 2006; Britto et al., 2017).

Respiratory viruses primarily infect and replicate within airway epithelial cells (Costa et al., 2014). During the replication process, the cells release antiviral factors and cytokines that alter local airway inflammation and airway niche (Busse et al., 2010). In a healthy airway, the inflammation normally leads to type 1 inflammatory responses consisting of activation of an antiviral state and infiltration of antiviral effector cells. This eventually results in the resolution of the inflammatory response and clearance of the viral infection (Vareille et al., 2011; Braciale et al., 2012). However, in a chronically inflamed airway, the responses against the virus may be impaired or aberrant, causing sustained inflammation and erroneous infiltration, resulting in the exacerbation of their symptoms (Mallia and Johnston, 2006; Dougherty and Fahy, 2009; Busse et al., 2010; Britto et al., 2017; Linden et al., 2019). This is usually further compounded by the increased susceptibility of chronic airway inflammatory disease patients toward viral respiratory infections, thereby increasing the frequency of exacerbation as a whole (Dougherty and Fahy, 2009; Busse et al., 2010; Linden et al., 2019).

Furthermore, due to the different replication cycles and response against the myriad of respiratory viruses, each respiratory virus may also contribute to exacerbations via different mechanisms that may alter their severity. Hence, this review will focus on compiling and collating the current known mechanisms of viral-induced exacerbation of chronic airway inflammatory diseases; as well as linking the different viral infection pathogenesis to elucidate other potential ways the infection can exacerbate the disease. The review will serve to provide further understanding of viral induced exacerbation to identify potential pathways and pathogenesis mechanisms that may be targeted as supplementary care for management and prevention of exacerbation. Such an approach may be clinically significant due to the current scarcity of antiviral drugs for the management of viral-induced exacerbations. This will improve the quality of life of patients with chronic airway inflammatory diseases.

## Current Understanding of Viral Induced Exacerbation of Chronic Airway Inflammatory Disease

Once the link between viral infection and acute exacerbations of chronic airway inflammatory disease was established, there have been many reports on the mechanisms underlying the exacerbation induced by respiratory viral infection. Upon infecting the host, viruses evoke an inflammatory response as a means of counteracting the infection. Generally, infected airway epithelial cells release type I (IFNα/β) and type III (IFNλ) interferons, cytokines and chemokines such as IL-6, IL-8, IL-12, RANTES, macrophage inflammatory protein 1α (MIP-1α) and monocyte chemotactic protein 1 (MCP-1) (Wark and Gibson, 2006; Matsukura et al., 2013). These, in turn, enable infiltration of innate immune cells and of professional antigen presenting cells (APCs) that will then in turn release specific mediators to facilitate viral targeting and clearance, including type II interferon (IFNγ), IL-2, IL-4, IL-5, IL-9, and IL-12 (Wark and Gibson, 2006; Singh et al., 2010; Braciale et al., 2012). These factors heighten local inflammation and the infiltration of granulocytes, T-cells and B-cells (Wark and Gibson, 2006; Braciale et al., 2012). The increased inflammation, in turn, worsens the symptoms of airway diseases.

Additionally, in patients with asthma and patients with CRS with nasal polyp (CRSwNP), viral infections such as RV and RSV promote a Type 2-biased immune response (Becker, 2006; Jackson et al., 2014; Jurak et al., 2018). This amplifies the basal type 2 inflammation resulting in a greater release of IL-4, IL-5, IL-13, RANTES and eotaxin and a further increase in eosinophilia, a key pathological driver of asthma and CRSwNP (Wark and Gibson, 2006; Singh et al., 2010; Chung et al., 2015; Dunican and Fahy, 2015). Increased eosinophilia, in turn, worsens the classical symptoms of disease and may further lead to life-threatening conditions due to breathing difficulties. On the other hand, patients with COPD and patients with CRS without nasal polyp (CRSsNP) are more neutrophilic in nature due to the expression of neutrophil chemoattractants such as CXCL9, CXCL10, and CXCL11 (Cukic et al., 2012; Brightling and Greening, 2019). The pathology of these airway diseases is characterized by airway remodeling due to the presence of remodeling factors such as matrix metalloproteinases (MMPs) released from infiltrating neutrophils (Linden et al., 2019). Viral infections in such conditions will then cause increase neutrophilic activation; worsening the symptoms and airway remodeling in the airway thereby exacerbating COPD, CRSsNP and even CRSwNP in certain cases (Wang et al., 2009; Tacon et al., 2010; Linden et al., 2019).

**FIGURE 1:**
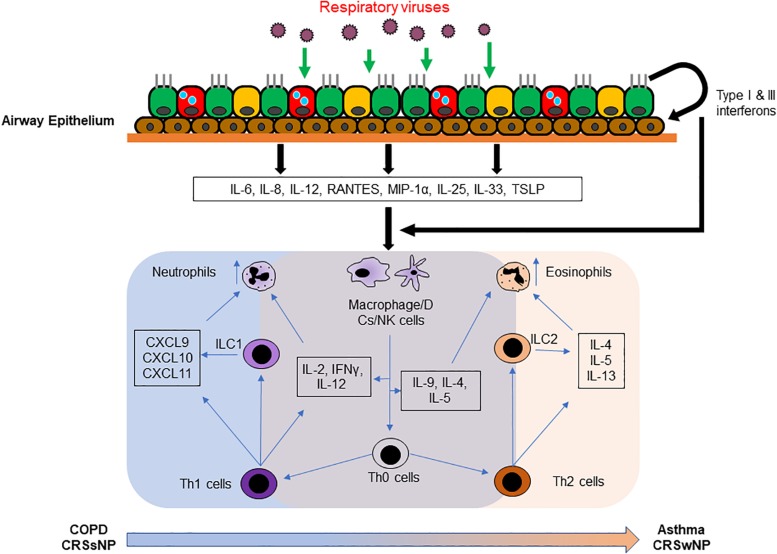
Current understanding of viral induced exacerbation of chronic airway inflammatory diseases. Upon virus infection in the airway, antiviral state will be activated to clear the invading pathogen from the airway. Immune response and injury factors released from the infected epithelium normally would induce a rapid type 1 immunity that facilitates viral clearance. However, in the inflamed airway, the cytokines and chemokines released instead augmented the inflammation present in the chronically inflamed airway, strengthening the neutrophilic infiltration in COPD airway, and eosinophilic infiltration in the asthmatic airway. The effect is also further compounded by the participation of Th1 and ILC1 cells in the COPD airway; and Th2 and ILC2 cells in the asthmatic airway.

An epithelial-centric alarmin pathway around IL-25, IL-33 and thymic stromal lymphopoietin (TSLP), and their interaction with group 2 innate lymphoid cells (ILC2) has also recently been identified (Nagarkar et al., 2012; Hong et al., 2018; Allinne et al., 2019). IL-25, IL-33 and TSLP are type 2 inflammatory cytokines expressed by the epithelial cells upon injury to the epithelial barrier (Gabryelska et al., 2019; Roan et al., 2019). ILC2s are a group of lymphoid cells lacking both B and T cell receptors but play a crucial role in secreting type 2 cytokines to perpetuate type 2 inflammation when activated (Scanlon and McKenzie, 2012; Li and Hendriks, 2013). In the event of viral infection, cell death and injury to the epithelial barrier will also induce the expression of IL-25, IL-33 and TSLP, with heighten expression in an inflamed airway (Allakhverdi et al., 2007; Goldsmith et al., 2012; Byers et al., 2013; Shaw et al., 2013; Beale et al., 2014; Jackson et al., 2014; Uller and Persson, 2018; Ravanetti et al., 2019). These 3 cytokines then work in concert to activate ILC2s to further secrete type 2 cytokines IL-4, IL-5, and IL-13 which further aggravate the type 2 inflammation in the airway causing acute exacerbation (Camelo et al., 2017). In the case of COPD, increased ILC2 activation, which retain the capability of differentiating to ILC1, may also further augment the neutrophilic response and further aggravate the exacerbation (Silver et al., 2016). Interestingly, these factors are not released to any great extent and do not activate an ILC2 response during viral infection in healthy individuals (Yan et al., 2016; Tan et al., 2018a); despite augmenting a type 2 exacerbation in chronically inflamed airways (Jurak et al., 2018). These classical mechanisms of viral induced acute exacerbations are summarized in Figure 1.

## Novel Mechanisms in the Upper Airway Hypothesized to Contribute to Viral Induced Acute Exacerbations

As integration of the virology, microbiology and immunology of viral infection becomes more interlinked, additional factors and mechanisms have been implicated in acute exacerbations during and after viral infection (Murray et al., 2006). Murray et al. (2006) has underlined the synergistic effect of viral infection with other sensitizing agents in causing more severe acute exacerbations in the airway. This is especially true when not all exacerbation events occurred during the viral infection but may also occur well after viral clearance (Kim et al., 2008; Stolz et al., 2019) in particular the late onset of a bacterial infection (Singanayagam et al., 2018, 2019a). In addition, viruses do not need to directly infect the lower airway to cause an acute exacerbation, as the nasal epithelium remains the primary site of most infections. Moreover, not all viral infections of the airway will lead to acute exacerbations, suggesting a more complex interplay between the virus and upper airway epithelium which synergize with the local airway environment in line with the “united airway” hypothesis (Kurai et al., 2013). On the other hand, viral infections or their components persist in patients with chronic airway inflammatory disease (Kling et al., 2005; Wood et al., 2011; Ravi et al., 2019). Hence, their presence may further alter the local environment and contribute to current and future exacerbations. Future studies should be performed using metagenomics in addition to PCR analysis to determine the contribution of the microbiome and mycobiome to viral infections. In this review, we highlight recent data regarding viral interactions with the airway epithelium that could also contribute to, or further aggravate, acute exacerbations of chronic airway inflammatory diseases.

### Increase Viral Susceptibility and Prolong Activation of Inflammation

Patients with chronic airway inflammatory diseases have impaired or reduced ability of viral clearance (Hammond et al., 2015; McKendry et al., 2016; Akbarshahi et al., 2018; Gill et al., 2018; Wang et al., 2018; Singanayagam et al., 2019b). Their impairment stems from a type 2-skewed inflammatory response which deprives the airway of important type 1 responsive CD8 cells that are responsible for the complete clearance of virus-infected cells (Becker, 2006; McKendry et al., 2016). This is especially evident in weak type 1 inflammation-inducing viruses such as RV and RSV (Kling et al., 2005; Wood et al., 2011; Ravi et al., 2019). Additionally, there are also evidence of reduced type I (IFNβ) and III (IFNλ) interferon production due to type 2-skewed inflammation, which contributes to imperfect clearance of the virus resulting in persistence of viral components, or the live virus in the airway epithelium (Contoli et al., 2006; Hwang et al., 2019; Wark, 2019). Due to the viral components remaining in the airway, antiviral genes such as type I interferons, inflammasome activating factors and cytokines remained activated resulting in prolong airway inflammation (Wood et al., 2011; Essaidi-Laziosi et al., 2018). These factors enhance granulocyte infiltration thus prolonging the exacerbation symptoms. Such persistent inflammation may also be found within DNA viruses such as AdV, hCMV and HSV, whose infections generally persist longer (Imperiale and Jiang, 2015), further contributing to chronic activation of inflammation when they infect the airway (Yang et al., 2008; Morimoto et al., 2009; Imperiale and Jiang, 2015; Lan et al., 2016; Tan et al., 2016; Kowalski et al., 2017). With that note, human papilloma virus (HPV), a DNA virus highly associated with head and neck cancers and respiratory papillomatosis, is also linked with the chronic inflammation that precedes the malignancies (de Visser et al., 2005; Gillison et al., 2012; Bonomi et al., 2014; Fernandes et al., 2015). Therefore, the role of HPV infection in causing chronic inflammation in the airway and their association to exacerbations of chronic airway inflammatory diseases, which is scarcely explored, should be investigated in the future. Furthermore, viral persistence which lead to continuous expression of antiviral genes may also lead to the development of steroid resistance, which is seen with RV, RSV, and PIV infection (Chi et al., 2011; Ford et al., 2013; Papi et al., 2013). The use of steroid to suppress the inflammation may also cause the virus to linger longer in the airway due to the lack of antiviral clearance (Kim et al., 2008; Hammond et al., 2015; Hewitt et al., 2016; McKendry et al., 2016; Singanayagam et al., 2019b). The concomitant development of steroid resistance together with recurring or prolong viral infection thus added considerable burden to the management of acute exacerbation, which should be the future focus of research to resolve the dual complications arising from viral infection.

### Destruction of the Epithelial Barrier

On the other end of the spectrum, viruses that induce strong type 1 inflammation and cell death such as IFV (Yan et al., 2016; Guibas et al., 2018) and certain CoV (including the recently emerged COVID-19 virus) (Tao et al., 2013; Yue et al., 2018; Zhu et al., 2020), may not cause prolonged inflammation due to strong induction of antiviral clearance. These infections, however, cause massive damage and cell death to the epithelial barrier, so much so that areas of the epithelium may be completely absent post infection (Yan et al., 2016; Tan et al., 2019). Factors such as RANTES and CXCL10, which recruit immune cells to induce apoptosis, are strongly induced from IFV infected epithelium (Ampomah et al., 2018; Tan et al., 2019). Additionally, necroptotic factors such as RIP3 further compounds the cell deaths in IFV infected epithelium (Tan et al., 2019). The massive cell death induced may result in worsening of the acute exacerbation due to the release of their cellular content into the airway, further evoking an inflammatory response in the airway (Guibas et al., 2018). Moreover, the destruction of the epithelial barrier may cause further contact with other pathogens and allergens in the airway which may then prolong exacerbations or results in new exacerbations. Epithelial destruction may also promote further epithelial remodeling during its regeneration as viral infection induces the expression of remodeling genes such as MMPs and growth factors (Tan et al., 2017). Infections that cause massive destruction of the epithelium, such as IFV, usually result in severe acute exacerbations with non-classical symptoms of chronic airway inflammatory diseases. Fortunately, annual vaccines are available to prevent IFV infections (Vasileiou et al., 2017; Zheng et al., 2018); and it is recommended that patients with chronic airway inflammatory disease receive their annual influenza vaccination as the best means to prevent severe IFV induced exacerbation.

### Augmentation of Infiltration by Increasing Barrier Leakiness

Another mechanism that viral infections may use to drive acute exacerbations is the induction of vasodilation or tight junction opening factors which may increase the rate of infiltration. Infection with a multitude of respiratory viruses causes disruption of tight junctions with the resulting increased rate of viral infiltration. This also increases the chances of allergens coming into contact with airway immune cells. For example, IFV infection was found to induce oncostatin M (OSM) which causes tight junction opening (Pothoven et al., 2015; Tian et al., 2018). Similarly, RV and RSV infections usually cause tight junction opening which may also increase the infiltration rate of eosinophils and thus worsening of the classical symptoms of chronic airway inflammatory diseases (Sajjan et al., 2008; Kast et al., 2017; Kim et al., 2018). In addition, the expression of vasodilating factors and fluid homeostatic factors such as angiopoietin-like 4 (ANGPTL4) and bactericidal/permeability-increasing fold-containing family member A1 (BPIFA1) are also associated with viral infections and pneumonia development, which may worsen inflammation in the lower airway (Li et al., 2015; Akram et al., 2018). These factors may serve as targets to prevent viral-induced exacerbations during the management of acute exacerbation of chronic airway inflammatory diseases.

### Alteration of Airway Microbiome

Another recent area of interest is the relationship between asthma and COPD exacerbations and their association with the airway microbiome. The development of chronic airway inflammatory diseases is usually linked to specific bacterial species in the microbiome which may thrive in the inflamed airway environment (Diver et al., 2019). In the event of a viral infection such as RV infection, the effect induced by the virus may destabilize the equilibrium of the microbiome present (Molyneaux et al., 2013; Kloepfer et al., 2014; Kloepfer et al., 2017; Jubinville et al., 2018; van Rijn et al., 2019). In addition, viral infection may disrupt biofilm colonies in the upper airway (e.g., *Streptococcus pneumoniae*) microbiome to be release into the lower airway and worsening the inflammation (Marks et al., 2013; Chao et al., 2014). Moreover, a viral infection may also alter the nutrient profile in the airway through release of previously inaccessible nutrients that will alter bacterial growth (Siegel et al., 2014; Mallia et al., 2018). Furthermore, the destabilization is further compounded by impaired bacterial immune response, either from direct viral influences, or use of corticosteroids to suppress the exacerbation symptoms (Singanayagam et al., 2018, 2019a; Wang et al., 2018; Finney et al., 2019). All these may gradually lead to more far reaching effect when normal flora is replaced with opportunistic pathogens, altering the inflammatory profiles (Teo et al., 2018). These changes may in turn result in more severe and frequent acute exacerbations due to the interplay between virus and pathogenic bacteria in exacerbating chronic airway inflammatory diseases (Wark et al., 2013; Singanayagam et al., 2018). To counteract these effects, microbiome-based therapies are in their infancy but have shown efficacy in the treatments of irritable bowel syndrome by restoring the intestinal microbiome (Bakken et al., 2011). Further research can be done similarly for the airway microbiome to be able to restore the microbiome following disruption by a viral infection.

### Disruption of Mucocilary Functions and Balance

Viral infections can cause the disruption of mucociliary function, an important component of the epithelial barrier. Ciliary proteins that aid in the proper function of the motile cilia in the airways are aberrantly expressed in ciliated airway epithelial cells which are the major target for RV infection (Griggs et al., 2017). Such form of secondary cilia dyskinesia appears to be present with chronic inflammations in the airway, but the exact mechanisms are still unknown (Peng et al., 2018, 2019; Qiu et al., 2018). Nevertheless, it was found that in viral infection such as IFV, there can be a change in the metabolism of the cells as well as alteration in the ciliary gene expression, mostly in the form of down-regulation of the genes such as dynein axonemal heavy chain 5 (DNAH5) and multiciliate differentiation And DNA synthesis associated cell cycle protein (MCIDAS) (Tan et al., 2018b, 2019). The recently emerged Wuhan CoV was also found to reduce ciliary beating in infected airway epithelial cell model (Zhu et al., 2020). Furthermore, viral infections such as RSV was shown to directly destroy the cilia of the ciliated cells and almost all respiratory viruses infect the ciliated cells (Jumat et al., 2015; Yan et al., 2016; Tan et al., 2018a). In addition, mucus overproduction may also disrupt the equilibrium of the mucociliary function following viral infection, resulting in symptoms of acute exacerbation (Zhu et al., 2009). Hence, the disruption of the ciliary movement during viral infection may cause more foreign material and allergen to enter the airway, aggravating the symptoms of acute exacerbation and making it more difficult to manage. The mechanism of the occurrence of secondary cilia dyskinesia can also therefore be explored as a means to limit the effects of viral induced acute exacerbation.

### miRNA and Other Epigenetic Modulation of Inflammation

MicroRNAs (miRNAs) are short non-coding RNAs involved in post-transcriptional modulation of biological processes, and implicated in a number of diseases (Tan et al., 2014). miRNAs are found to be induced by viral infections and may play a role in the modulation of antiviral responses and inflammation (Gutierrez et al., 2016; Deng et al., 2017; Feng et al., 2018). In the case of chronic airway inflammatory diseases, circulating miRNA changes were found to be linked to exacerbation of the diseases (Wardzynska et al., 2020). Therefore, it is likely that such miRNA changes originated from the infected epithelium and responding immune cells, which may serve to further dysregulate airway inflammation leading to exacerbations. Both IFV and RSV infections has been shown to increase miR-21 and augmented inflammation in experimental murine asthma models, which is reversed with a combination treatment of anti-miR-21 and corticosteroids (Kim et al., 2017). IFV infection is also shown to increase miR-125a and b, and miR-132 in COPD epithelium which inhibits A20 and MAVS; and p300 and IRF3, respectively, resulting in increased susceptibility to viral infections (Hsu et al., 2016, 2017). Conversely, miR-22 was shown to be suppressed in asthmatic epithelium in IFV infection which lead to aberrant epithelial response, contributing to exacerbations (Moheimani et al., 2018). Other than these direct evidence of miRNA changes in contributing to exacerbations, an increased number of miRNAs and other non-coding RNAs responsible for immune modulation are found to be altered following viral infections (Globinska et al., 2014; Feng et al., 2018; Hasegawa et al., 2018). Hence non-coding RNAs also presents as targets to modulate viral induced airway changes as a means of managing exacerbation of chronic airway inflammatory diseases. Other than miRNA modulation, other epigenetic modification such as DNA methylation may also play a role in exacerbation of chronic airway inflammatory diseases. Recent epigenetic studies have indicated the association of epigenetic modification and chronic airway inflammatory diseases, and that the nasal methylome was shown to be a sensitive marker for airway inflammatory changes (Cardenas et al., 2019; Gomez, 2019). At the same time, it was also shown that viral infections such as RV and RSV alters DNA methylation and histone modifications in the airway epithelium which may alter inflammatory responses, driving chronic airway inflammatory diseases and exacerbations (McErlean et al., 2014; Pech et al., 2018; Caixia et al., 2019). In addition, Spalluto et al. (2017) also showed that antiviral factors such as IFNγ epigenetically modifies the viral resistance of epithelial cells. Hence, this may indicate that infections such as RV and RSV that weakly induce antiviral responses may result in an altered inflammatory state contributing to further viral persistence and exacerbation of chronic airway inflammatory diseases (Spalluto et al., 2017).

### Oxidative Stress

**FIGURE 2:**
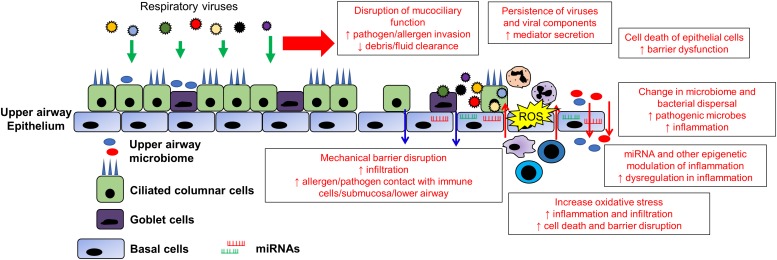
Changes in the upper airway epithelium contributing to viral exacerbation in chronic airway inflammatory diseases. The upper airway epithelium is the primary contact/infection site of most respiratory viruses. Therefore, its infection by respiratory viruses may have far reaching consequences in augmenting and synergizing current and future acute exacerbations. The destruction of epithelial barrier, mucociliary function and cell death of the epithelial cells serves to increase contact between environmental triggers with the lower airway and resident immune cells. The opening of tight junction increasing the leakiness further augments the inflammation and exacerbations. In addition, viral infections are usually accompanied with oxidative stress which will further increase the local inflammation in the airway. The dysregulation of inflammation can be further compounded by modulation of miRNAs and epigenetic modification such as DNA methylation and histone modifications that promote dysregulation in inflammation. Finally, the change in the local airway environment and inflammation promotes growth of pathogenic bacteria that may replace the airway microbiome. Furthermore, the inflammatory environment may also disperse upper airway commensals into the lower airway, further causing inflammation and alteration of the lower airway environment, resulting in prolong exacerbation episodes following viral infection.

Finally, viral infection can result in enhanced production of reactive oxygen species (ROS), oxidative stress and mitochondrial dysfunction in the airway epithelium (Kim et al., 2018; Mishra et al., 2018; Wang et al., 2018). The airway epithelium of patients with chronic airway inflammatory diseases are usually under a state of constant oxidative stress which sustains the inflammation in the airway (Barnes, 2017; van der Vliet et al., 2018). Viral infections of the respiratory epithelium by viruses such as IFV, RV, RSV and HSV may trigger the further production of ROS as an antiviral mechanism (Liu et al., 2017; To et al., 2017; Aizawa et al., 2018; Wang et al., 2018). Moreover, infiltrating cells in response to the infection such as neutrophils will also trigger respiratory burst as a means of increasing the ROS in the infected region. The increased ROS and oxidative stress in the local environment may serve as a trigger to promote inflammation thereby aggravating the inflammation in the airway (Tiwari et al., 2002). A summary of potential exacerbation mechanisms and the associated viruses is shown in Figure 2 and Table 1.

**TABLE 1:** Summary of literature evidence of potential viral induced exacerbation mechanisms in chronic airway inflammatory diseases at the upper airway epithelium.

**Types of exacerbation mechanism**	**Viral specific trait contributing to exacerbation mechanism (with literature evidence)**
Increased viral susceptibility and prolonged activation of inflammation	Weak type 1 inflammation leading to skewed type 2 inflammation (RV, RSV) Persistence of virus and viral components (RV, RSV, AdV, hCMV, HSV) Development of steroid resistance (RV, RSV, PIV)
Destruction of the epithelial barrier	Diffused cell death in the epithelial layer (IFV, CoV)
Augmentation of infiltration by increasing barrier leakiness	Disruption of tight junctions (RV, RSV) Oncostatin M induction (IFV) ANGPTL4 induction (IFV) BPIFA1 changes (IFV)
Alteration of airway microbiome	Destabilization of the microbiome (RV) Disruption of biofilm colonies (IFV) Alteration of the airway nutrient profile (RV, IFV) Reduced bacterial immunity (RV, possibly IFV and RSV)
Disruption of mucociliary functions and balance	Infection targeting ciliated cells (RV, IFV, RSV) Alteration of ciliary gene expression (IFV) Destruction of cilia and disruption of ciliary function (RSV, CoV) Mucus overproduction (RV)
miRNA and other epigenetic modulation of inflammation	miRNA modulation (IFV, RV, RSV) DNA methylation and histone modifications (RV, RSV)
Oxidative stress	ROS production (RV, RSV, IFV, HSV)

*As RV, RSV, and IFV were the most frequently studied viruses in chronic airway inflammatory diseases, most of the viruses listed are predominantly these viruses. However, the mechanisms stated here may also be applicable to other viruses but may not be listed as they were not implicated in the context of chronic airway inflammatory diseases exacerbation (see text for abbreviations).*

## Clinical Significance of Identifying Additional Mechanisms of Acute Exacerbations

While the mechanisms underlying the development and acute exacerbation of chronic airway inflammatory disease is extensively studied for ways to manage and control the disease, a viral infection does more than just causing an acute exacerbation in these patients. A viral-induced acute exacerbation not only induced and worsens the symptoms of the disease, but also may alter the management of the disease or confer resistance toward treatments that worked before. Hence, appreciation of the mechanisms of viral-induced acute exacerbations is of clinical significance to devise strategies to correct viral induce changes that may worsen chronic airway inflammatory disease symptoms. Further studies in natural exacerbations and in viral-challenge models using RNA-sequencing (RNA-seq) or single cell RNA-seq on a range of time-points may provide important information regarding viral pathogenesis and changes induced within the airway of chronic airway inflammatory disease patients to identify novel targets and pathway for improved management of the disease. Subsequent analysis of functions may use epithelial cell models such as the air-liquid interface, *in vitro* airway epithelial model that has been adapted to studying viral infection and the changes it induced in the airway (Yan et al., 2016; Boda et al., 2018; Tan et al., 2018a). Animal-based diseased models have also been developed to identify systemic mechanisms of acute exacerbation (Shin, 2016; Gubernatorova et al., 2019; Tanner and Single, 2019). Furthermore, the humanized mouse model that possess human immune cells may also serves to unravel the immune profile of a viral infection in healthy and diseased condition (Ito et al., 2019; Li and Di Santo, 2019). For milder viruses, controlled *in vivo* human infections can be performed for the best mode of verification of the associations of the virus with the proposed mechanism of viral induced acute exacerbations (Ravi et al., 2019). With the advent of suitable diseased models, the verification of the mechanisms will then provide the necessary continuation of improving the management of viral induced acute exacerbations.

## Conclusion and Future Outlook

In conclusion, viral-induced acute exacerbation of chronic airway inflammatory disease is a significant health and economic burden that needs to be addressed urgently. In view of the scarcity of antiviral-based preventative measures available for only a few viruses and vaccines that are only available for IFV infections, more alternative measures should be explored to improve the management of the disease. Alternative measures targeting novel viral-induced acute exacerbation mechanisms, especially in the upper airway, can serve as supplementary treatments of the currently available management strategies to augment their efficacy. New models including primary human bronchial or nasal epithelial cell cultures, organoids or precision cut lung slices from patients with airways disease rather than healthy subjects can be utilized to define exacerbation mechanisms. These mechanisms can then be validated in small clinical trials in patients with asthma or COPD. Having multiple means of treatment may also reduce the problems that arise from resistance development toward a specific treatment.

## Author Contributions

KT, VC, and DW contributed to the initial conceptualization of the manuscript. KT, RL, JL, HO, and VT contributed to literature search. KT, HL, JL, HO, VT, HL, IA, VC, and DW contributed to literature selection. KT, RL, IA, and VC contributed to the writing of manuscript. KT, RL, HL, KC, IA, VC, and DW contributed to the review and finalization of the manuscript.

## Conflict of Interest

The authors declare that the research was conducted in the absence of any commercial or financial relationships that could be construed as a potential conflict of interest.
